# Extensive cervicofacial-to-scrotal subcutaneous emphysema without pneumothorax following pediatric blunt thoracic trauma: a case report

**DOI:** 10.1186/s12887-026-07292-6

**Published:** 2026-07-04

**Authors:** Esubalew Mihiret Alemayehu, Mulualem Amare Woldemichael, Bedilu Zewdu Asmare

**Affiliations:** 1 Department of Pediatrics, College of Medicine and Health Sciences, Dire Dawa University, Dire Dawa, Ethiopia; 2 Department of Surgery, College of Medicine and Health Sciences, Wolkite University, Wolkite, Ethiopia; 3 Department of Radiology, College of Medicine and Health Sciences, Wolkite University, Wolkite, Ethiopia

**Keywords:** pediatric trauma, pneumomediastinum, subcutaneous emphysema, pneumoscrotum, Macklin effect, blunt chest trauma

## Abstract

**Background:**

Pneumomediastinum with subcutaneous emphysema is an uncommon consequence of pediatric blunt thoracic trauma. Inferior extension of air into the scrotum (pneumoscrotum) is particularly rare, especially in the absence of pneumothorax.

**Case presentation:**

A previously healthy 7-year-old boy presented 8 hours after sustaining blunt thoracic trauma from an ox kick directed to the left lower anterior chest. Swelling began within one hour and progressively extended from the face and neck to the chest, abdomen, and scrotum. Despite extensive cervicofacial, thoracoabdominal, and scrotal subcutaneous emphysema with diffuse crepitus, he remained hemodynamically stable without significant respiratory compromise. Hamman’s sign was present. Chest radiography demonstrated pneumomediastinum with the continuous diaphragm sign and extensive subcutaneous emphysema, without pneumothorax. Computed tomography confirmed pneumomediastinum with air tracking along bronchovascular sheaths consistent with the Macklin effect, retroperitoneal extension, and a focal pulmonary contusion. No tracheobronchial or esophageal injury was identified. The patient was managed conservatively with observation, analgesia, activity restriction, and supplemental oxygen. Complete resolution occurred within 7 days.

**Conclusions:**

Extensive subcutaneous emphysema with pneumoscrotum can occur following pediatric blunt thoracic trauma via the Macklin effect without pneumothorax. Recognition of this pattern and correlation with clinical stability allows safe conservative management and avoidance of unnecessary invasive interventions.

**Supplementary Information:**

The online version contains supplementary material available at https://doi.org/10.1186/s12887-026-07292-6.

## Background

Pneumomediastinum refers to the presence of free air within the mediastinum and may occur spontaneously or secondary to trauma, infection, or iatrogenic causes [1, 2]. In children, blunt thoracic trauma is a recognized cause, partly due to increased chest wall compliance, which allows transmission of kinetic energy to the pulmonary parenchyma without rib fractures [3, 4].

Subcutaneous emphysema commonly accompanies pneumomediastinum; however, inferior extension into the scrotum (pneumoscrotum) is exceedingly rare in pediatric patients [1, 5]. Air may dissect along fascial planes from the mediastinum into the retroperitoneum and subsequently into the scrotum via the inguinal canal [5, 6].

We report a case of extensive subcutaneous emphysema extending from the cervicofacial region to the scrotum, associated with pneumomediastinum without pneumothorax following pediatric blunt thoracic trauma.

## Case presentation

### Patient information

A previously healthy 7-year-old boy sustained blunt thoracic trauma caused by an ox kick directed to the left lower anterior chest. Approximately 1 h after the injury, he was taken by family members to a nearby health center, where he was assessed, placed on intranasal supplemental oxygen, and started on intravenous (IV) fluids. After approximately 4 to 5 h at the health center, he was referred to our hospital and transported by ambulance with supplemental oxygen and an intravenous line in situ, with the journey taking approximately 2 h, arriving at our emergency department a total of approximately 8 h after the initial trauma. Swelling had begun approximately 1 h after the injury and progressed over several hours, extending from the face to the neck, chest, abdomen, and scrotum. There was no history of loss of consciousness, vomiting, dysphagia, or pre-existing pulmonary disease.

## Clinical findings

### Vital signs

**Table:** 

Parameter	Value
Heart rate	90 beats/min
Respiratory rate	40 breaths/min
Blood pressure	100/60 mmHg
Oxygen saturation	95% (on supplemental oxygen)
Temperature	36.6 °C

**Fig. 1:**
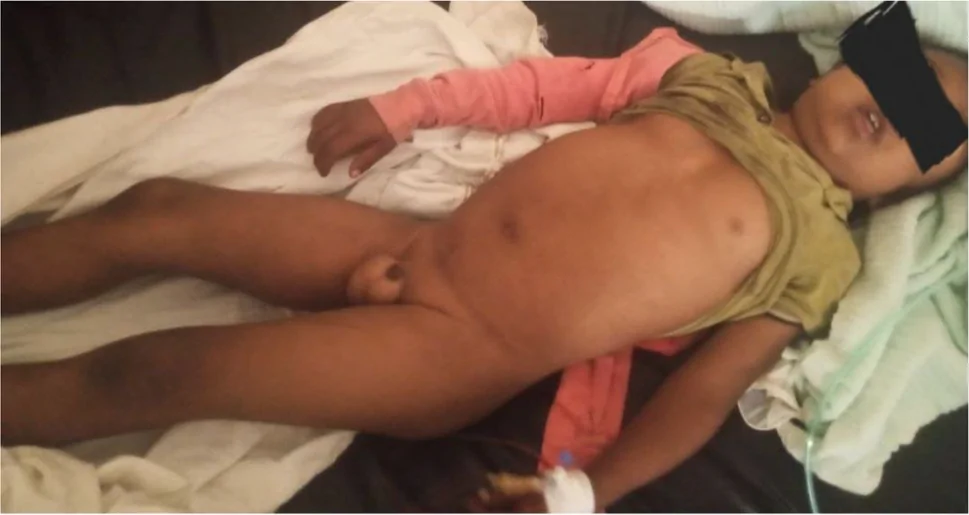
Clinical photograph demonstrating extensive subcutaneous emphysema involving the face, neck, abdominal wall, and scrotum with palpable crepitus in a 7-year-old boy following blunt thoracic trauma (ox kick injury)

The patient was alert and hemodynamically stable. Examination revealed marked swelling of the face, neck, chest, abdomen, and scrotum with diffuse crepitus on palpation. Maximal tenderness was elicited over the left anterior chest wall, just above the left subcostal margin, consistent with the reported site of impact. There was no visible laceration or degloving injury. Despite tachypnea, there were no signs of respiratory distress such as retractions, nasal flaring, or use of accessory muscles, and air entry was equal bilaterally. A precordial crunch synchronous with the heartbeat was present on auscultation, consistent with Hamman’s sign — a crunching or crackling sound heard over the precordium during systole, caused by mediastinal air compressing against cardiac structures and widely regarded as a clinical hallmark of pneumomediastinum (Fig. 1).

### Clinical timeline

**Table:** 

Day	Clinical Progress
Day 0	Presentation 8 h post-injury; initiation of supplemental oxygen via nasal prongs and analgesia.
Day 3	Significant improvement in cervicofacial emphysema.
Day 5	Complete resolution of pneumoscrotum.
Day 7	Complete resolution of all subcutaneous emphysema; patient asymptomatic.

### Diagnostic assessment

**Fig. 2:**
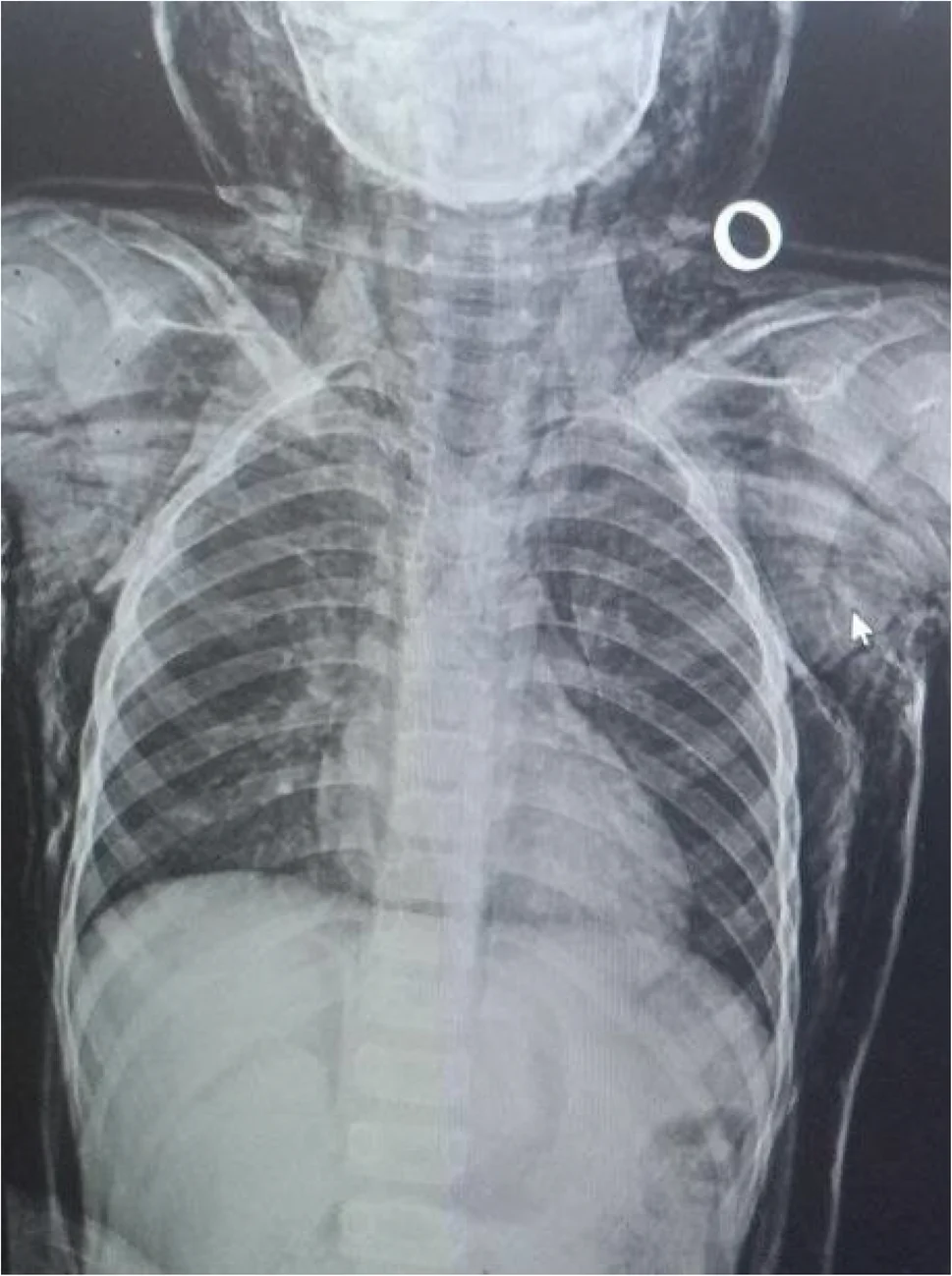
Posteroanterior chest radiograph demonstrating the continuous diaphragm sign, indicative of pneumomediastinum, and significant subcutaneous emphysema involving the neck and chest wall. No pneumothorax is identified

Chest radiography demonstrated the continuous diaphragm sign, indicative of pneumomediastinum, together with extensive subcutaneous emphysema involving the neck and chest wall. Importantly, no pneumothorax was identified (Fig. 2).

**Fig. 3:**
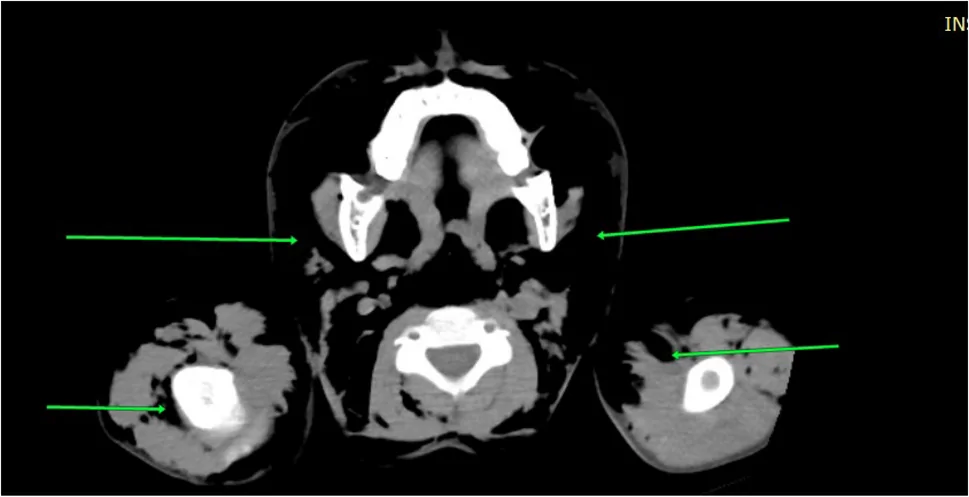
Axial CT at the level of the neck demonstrating widespread subcutaneous emphysema involving the neck, face, and bilateral upper limbs (arrows)

**Fig. 4:**
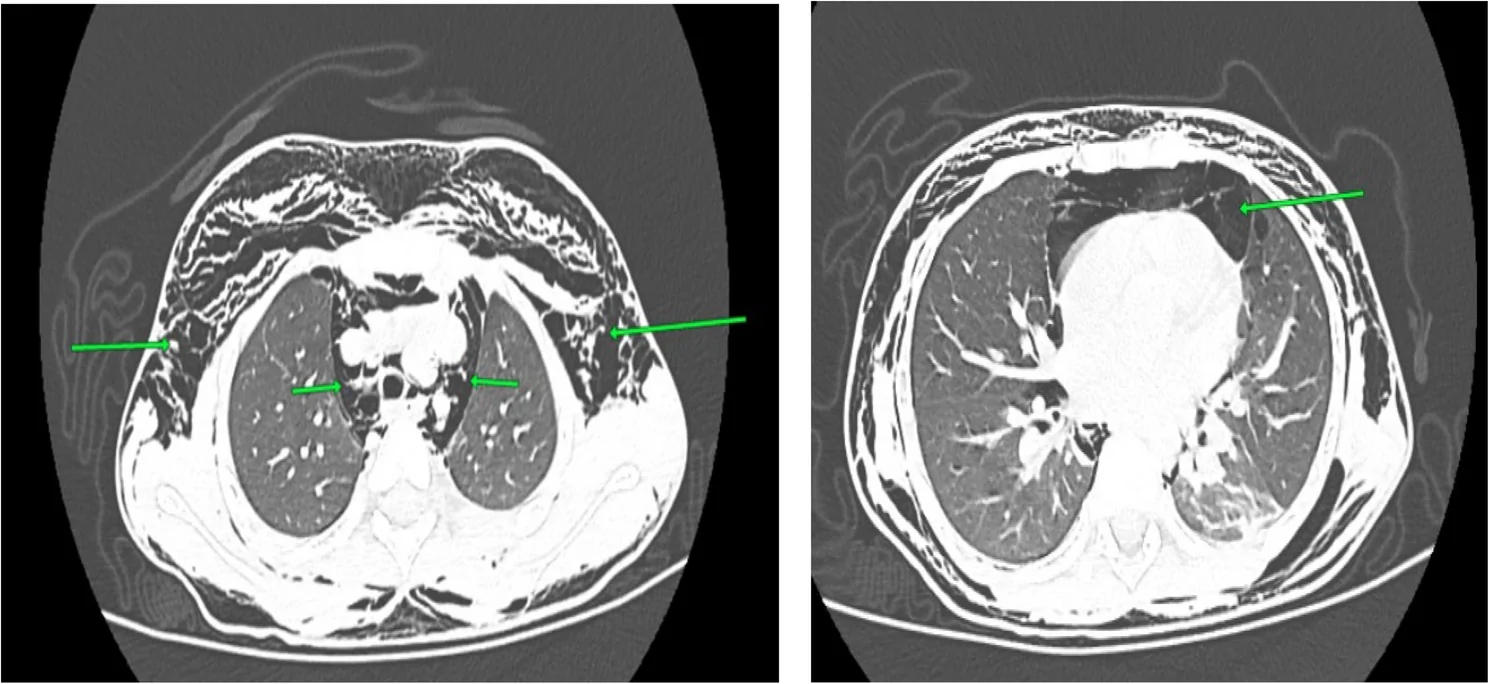
Axial chest CT demonstrating air outlining the mediastinal vessels, heart, trachea, and esophagus, consistent with extensive pneumomediastinum, together with chest wall subcutaneous emphysema (arrows)

**Fig. 5:**
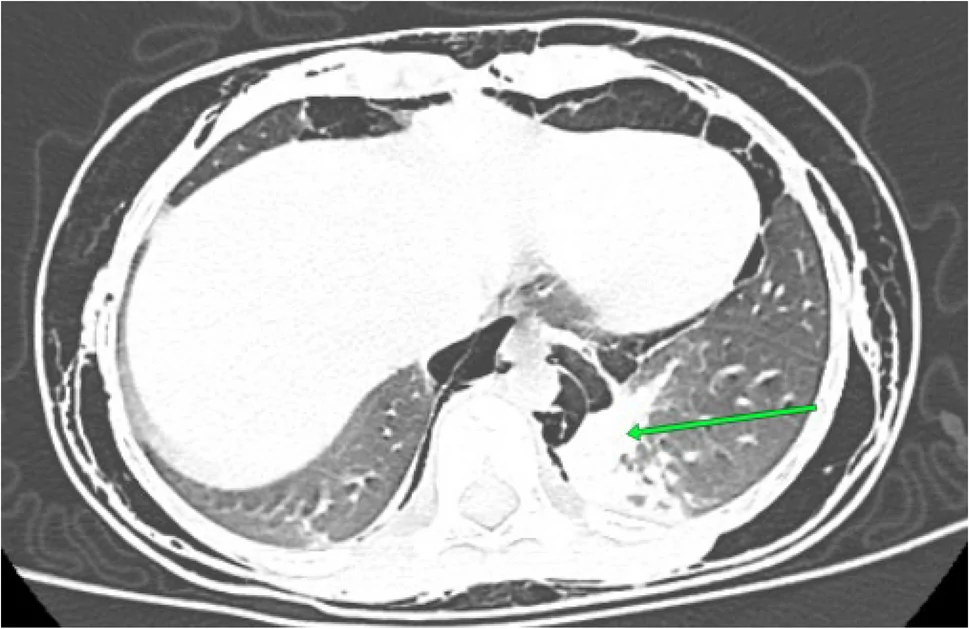
Axial chest CT demonstrating a focal air-space opacity in the left lower lobe consistent with pulmonary contusion (arrow). No pneumothorax is identified

**Fig. 6:**
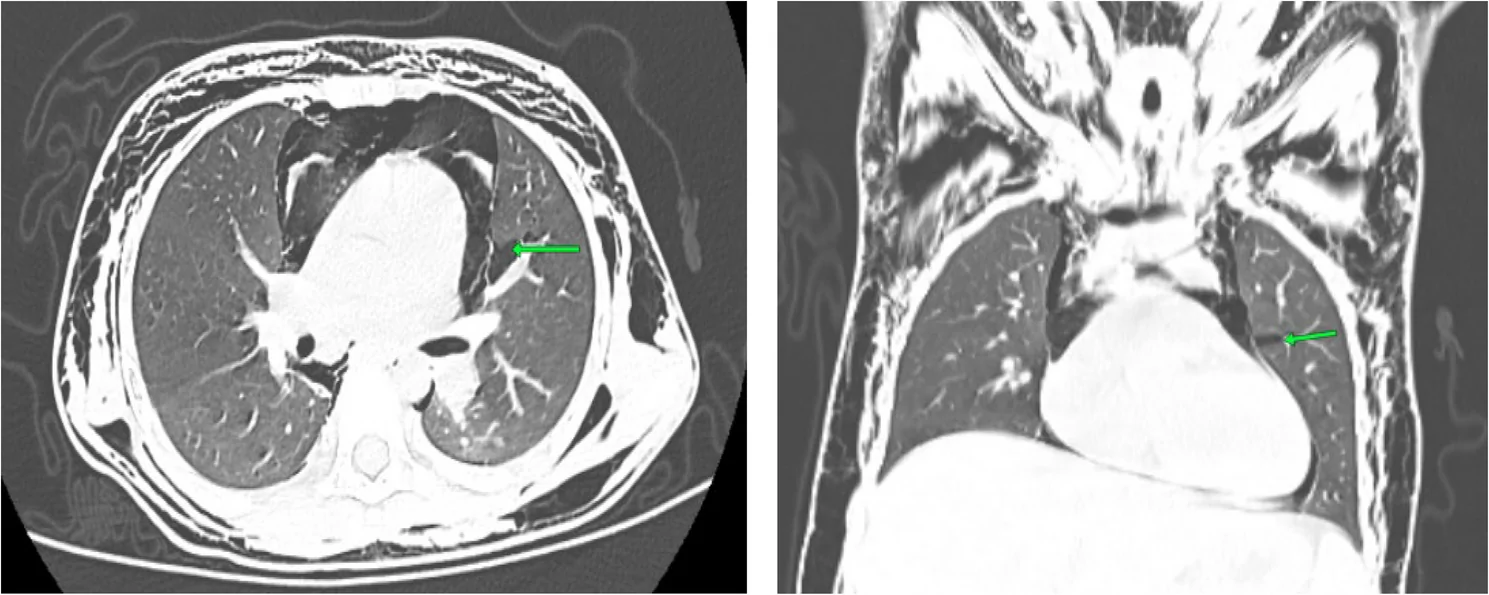
Axial and coronal chest CT demonstrating air outlining the axial pulmonary interstitium, confirming interstitial pulmonary emphysema (arrows). Both lungs remain fully expanded with no evidence of pneumothorax

**Fig. 7:**
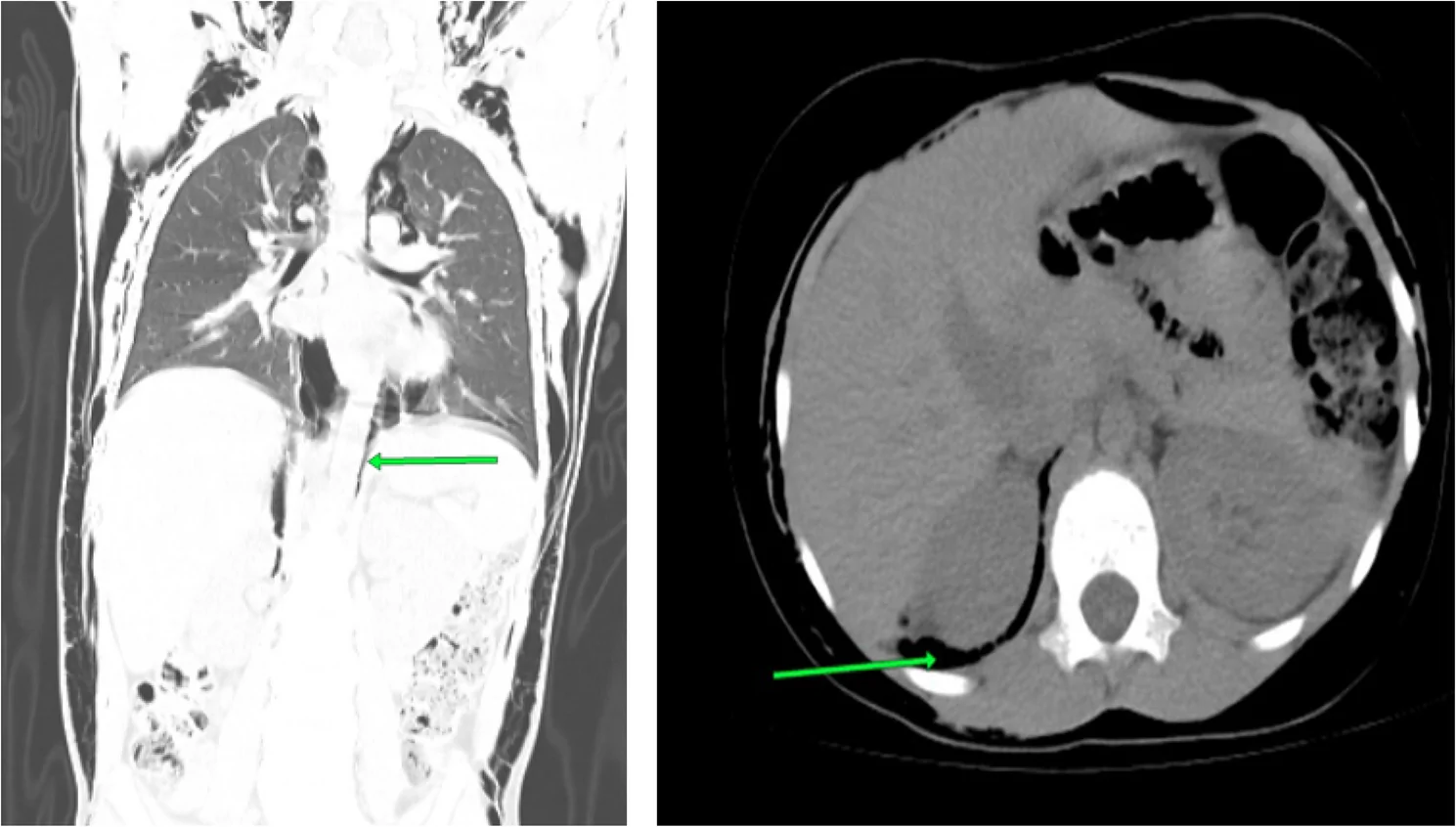
Coronal chest CT and axial abdominal CT demonstrating air tracking along the aorta from the mediastinum into the retroperitoneum, outlining the right kidney (arrows), consistent with retroperitoneal air dissection

Computed tomography (CT) of the chest and neck revealed extensive pneumomediastinum, with air outlining the trachea, esophagus, mediastinal vessels, and cardiac silhouette. Air tracking along bronchovascular sheaths into the mediastinum was consistent with the Macklin effect — the mechanism by which sudden increases in intra-alveolar pressure cause alveolar rupture, with air then dissecting along peribronchovascular connective tissue sheaths centrally into the mediastinum without breaching the visceral pleura, thereby producing pneumomediastinum in the absence of pneumothorax. Inferior extension of air was observed along the aorta into the retroperitoneum, with the right kidney outlined by retroperitoneal air (Figs. 3, 4, 5, 6 and 7). Interstitial pulmonary emphysema was evident, and a focal air-space opacity in the left lower lobe was consistent with pulmonary contusion. Widespread subcutaneous emphysema involved the bilateral upper limbs, neck, chest wall, and abdominal wall. No radiological evidence of tracheobronchial or esophageal injury was identified, and no rib fractures were seen. Laboratory investigations were unremarkable.

### Differential diagnosis

The following diagnoses were considered and systematically evaluated:

1. Traumatic pneumomediastinum via the Macklin effect — most consistent with clinical and radiological findings
2. Tracheobronchial injury — excluded by CT and clinical stability
3. Esophageal perforation — excluded by CT and absence of dysphagia or fever
4. Occult pneumothorax — excluded by CT
5. Gas-forming infection — excluded by normal laboratory indices and clinical course

### Therapeutic intervention

The patient was managed conservatively with:

1. Continuous observation and serial clinical examinations
2. Analgesia for pain management
3. Activity restriction
4. Supplemental oxygen via nasal prongs — administered primarily to support respiration in the context of tachypnea and pain-related splinting, and secondarily to promote resorption of mediastinal and subcutaneous air

No invasive procedures were performed.

### Follow-up and outcomes

**Fig. 8:**
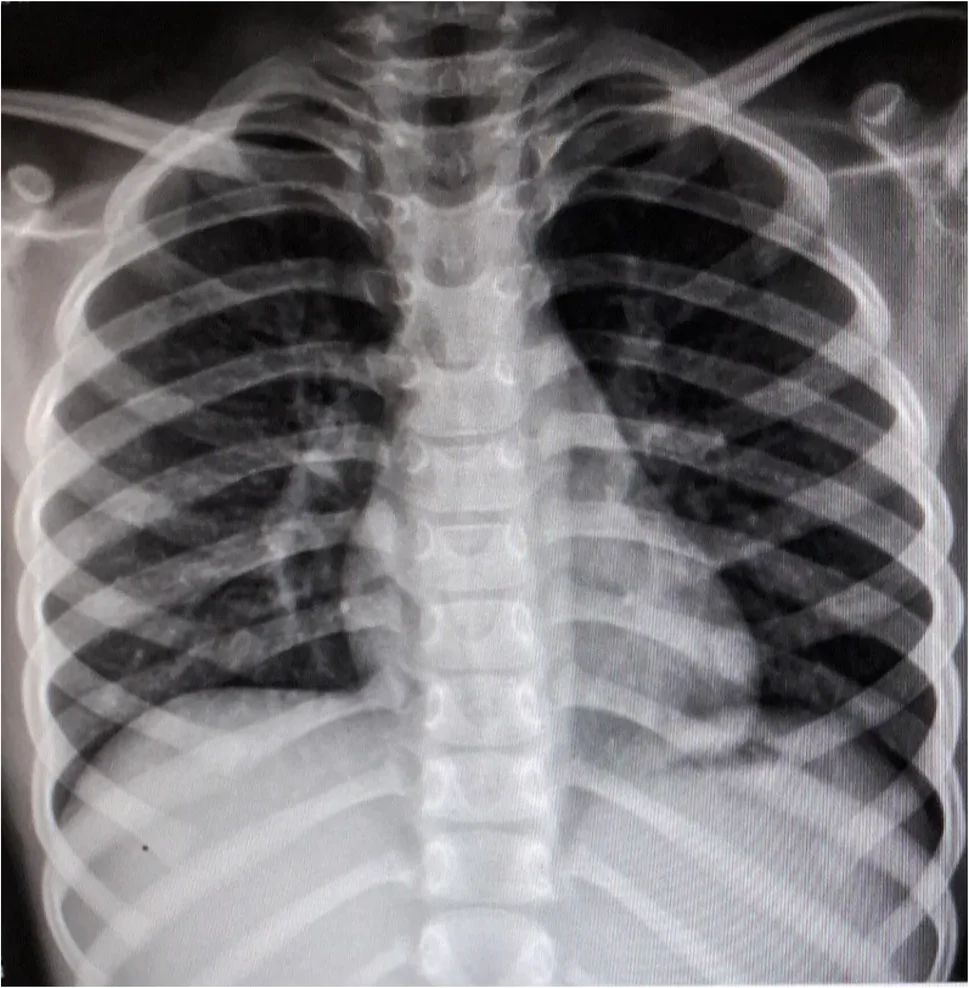
Posteroanterior chest radiograph obtained at the time of discharge (Day 7), demonstrating complete resolution of pneumomediastinum and subcutaneous emphysema. The continuous diaphragm sign is no longer present, and the mediastinal contour has returned to normal. Both lungs remain fully expanded with no pneumothorax

Cervicofacial emphysema showed significant improvement by day 3. Thoracoabdominal emphysema resolved over the subsequent days. Pneumoscrotum resolved completely by day 5. All subcutaneous emphysema had fully resolved by day 7, at which point the patient was asymptomatic. He remained well at outpatient follow-up with no recurrence (Fig. 8).

## Discussion

This case illustrates an uncommon but clinically significant manifestation of pediatric blunt thoracic trauma: extensive subcutaneous emphysema extending from the cervicofacial region to the scrotum, associated with pneumomediastinum in the absence of pneumothorax or aerodigestive injury. The striking extent of air dissemination stood in marked contrast to the patient’s preserved physiological stability, highlighting a well-recognized clinico-radiological dissociation in such cases.

The most plausible underlying mechanism is the Macklin effect, in which alveolar rupture leads to dissection of air along peribronchovascular sheaths into the mediastinum [7]. In pediatric patients, increased chest wall compliance facilitates transmission of kinetic force to the pulmonary parenchyma without producing rib fractures, predisposing to this mechanism [3]. Preservation of pleural integrity accounts for the absence of pneumothorax despite significant alveolar and interstitial injury [1]. CT findings in this case including air tracking along bronchovascular sheaths and interstitial pulmonary emphysema, directly confirmed the Macklin effect. Similar patterns with favorable outcomes have been described in pediatric traumatic pneumomediastinum [8].

Inferior spread of air into the scrotum is explained by the anatomical continuity of fascial planes, allowing air to dissect from the mediastinum into the retroperitoneum and through the inguinal canal into the scrotum [6]. Although pneumoscrotum is more commonly associated with intra-abdominal pathology, a thoracic source must be considered when the clinical context is consistent [5].

The primary clinical challenge lies in distinguishing benign pneumomediastinum from life-threatening aerodigestive injury. However, in hemodynamically stable patients without clinical features suggesting aerodigestive injury—such as dysphagia, fever, or progressive respiratory deterioration—conservative management is appropriate and supported by current evidence. Many cases of pneumomediastinum follow a benign, self-limiting course amenable to conservative management [2, 9].

Management in this case was entirely supportive. Supplemental oxygen was administered via nasal prongs for two complementary indications. The primary indication was respiratory support: the patient presented with tachypnea and pain-related splinting that risked compromising ventilation, making supplemental oxygen an immediate clinical necessity irrespective of the pneumomediastinum itself. The secondary benefit is the well-established role of oxygen therapy in accelerating resorption of ectopic air: by enriching the inspired oxygen fraction, a nitrogen concentration gradient is created across the alveolar-capillary membrane, which promotes diffusion of nitrogen out of the trapped air spaces and thereby hastens resolution of both pneumomediastinum and subcutaneous emphysema in clinically stable patients [10]. Current evidence supports a selective, non-interventional approach in stable patients, reserving invasive procedures for clinical deterioration or confirmed aerodigestive injury [9, 11–13]. This case reinforces the importance of clinical status rather than radiological extent as the primary guide to management in pediatric traumatic pneumomediastinum. In contrast to cases complicated by tension pneumomediastinum, progressive respiratory compromise, or confirmed aerodigestive injury, this patient’s preserved hemodynamic stability supported a non-interventional approach.

This report has several limitations. First, bronchoscopy and contrast esophagography were not performed; although the patient’s clinical stability and CT findings made aerodigestive injury unlikely, these investigations would have provided definitive exclusion. Second, follow-up was limited to the immediate post-discharge period, so late sequelae such as recurrence or subtle pulmonary impairment cannot be excluded.

## Conclusion

Extensive subcutaneous emphysema with pneumoscrotum can occur following pediatric blunt thoracic trauma without pneumothorax, mediated by the Macklin effect and air dissection along continuous fascial planes. Recognition of this clinico-radiological pattern allows for appropriate risk stratification, targeted exclusion of serious aerodigestive injury with CT, and safe conservative management. In resource-limited settings where CT availability may be restricted, hemodynamic stability and the absence of clinical features suggesting tracheobronchial or esophageal injury remain the most important guides to avoiding unnecessary invasive interventions.

### Why should a clinician be aware of this?

Extensive subcutaneous emphysema in a child following blunt thoracic trauma can appear alarming and may prompt urgent invasive investigation or intervention. However, this case illustrates that even dramatic craniocaudal air dissemination, extending from the face to the scrotum can occur via the Macklin effect without underlying pneumothorax or aerodigestive injury, and may follow a benign, self-limiting course with full resolution under conservative management.

Clinicians should be aware that pneumoscrotum, though more commonly associated with intra-abdominal pathology, can originate from thoracic air tracking through continuous fascial planes. Failure to recognize this pathway may lead to unnecessary urological or surgical investigations.

In resource-limited settings where CT availability may be restricted, clinical stability remains the most important guide to management. In a hemodynamically stable child without signs of respiratory distress or features suggesting esophageal or tracheobronchial injury, a conservative approach is appropriate and effective. Management decisions driven by the radiological extent of emphysema rather than clinical status risk exposing children to unnecessary procedures and radiation.

### Learning points

- Extensive subcutaneous emphysema does not always indicate severe or life-threatening injury.
- Pneumoscrotum can originate from thoracic air dissection along continuous fascial planes and should not be attributed reflexively to intra-abdominal pathology.
- The Macklin effect is the primary mechanism of traumatic pneumomediastinum in children and can occur without pneumothorax when pleural integrity is preserved.
- Clinical stability rather than radiological extent should guide management decisions.
- Conservative treatment with observation, analgesia, activity restriction, and supplemental oxygen via nasal prongs is effective in selected hemodynamically stable patients.

### Patient perspective

The patient’s father expressed surprise and relief at the child’s full recovery without invasive intervention. He reported satisfaction with the care provided and the outcome achieved through conservative management.

## Supplementary Information

Supplementary Material 1.

## Data Availability

No datasets were generated or analysed during the current study.
