# Supplementary material for: Extensive cervicofacial-to-scrotal subcutaneous emphysema without pneumothorax following pediatric blunt thoracic trauma: a case report
Source: BMC Pediatr. 2026 Jul 4;26:939. doi: 10.1186/s12887-026-07292-6 (PMC13637163; doi:10.1186/s12887-026-07292-6)
Supplement: Supplementary file 1 [file 12887_2026_7292_MOESM1_ESM.pdf]

Supplementary File 1. Care Guideline Checklist for Pediatric Traumatic Pneumomediastinum With Extensive Subcutaneous Emphysema

Table S1. Care guideline checklist for assessment and management

Domain	Checklist items
1. Initial assessment (Emergency Department)	☐Confirm mechanism, timing, and force of blunt thoracic trauma<br>☐Airway: voice change, stridor, hoarseness<br>☐Breathing: respiratory rate, pattern, oxygen saturation, signs of distress<br>☐Circulation: heart rate, blood pressure, capillary refill<br>☐Neurological status (AVPU/GCS)<br>☐Full exposure to assess extent of subcutaneous emphysema
2. Focused clinical examination	☐Palpable crepitus; document extent (face, neck, chest, abdomen, scrotum)<br>☐Chest auscultation: symmetric air entry<br>☐Hamman's sign<br>☐Screen for aerodigestive symptoms<br>☐Abdominal and scrotal examination
3. Initial investigations	☐Chest radiograph (AP/PA)<br>☐Identify pneumomediastinum (e.g., continuous diaphragm sign)<br>☐Assess subcutaneous emphysema<br>☐Determine presence or absence of pneumothorax<br>☐Baseline laboratory tests if indicated
4. Indications for CT imaging	☐Extensive or progressive subcutaneous emphysema<br>☐Inconclusive chest radiograph<br>☐Suspected tracheobronchial injury<br>☐Suspected esophageal injury<br>☐Unexplained or worsening respiratory compromise
5. CT assessment checklist	☐Confirm pneumomediastinum<br>☐Air tracking along bronchovascular sheaths (Macklin effect)<br>☐Presence or absence of pneumothorax<br>☐Pulmonary contusion or interstitial emphysema<br>☐Retroperitoneal air extension<br>☐Exclusion of tracheobronchial and esophageal injury
6. Risk stratification	Low-risk:<br>☐Hemodynamically stable<br>☐No significant respiratory distress<br>☐No or small stable pneumothorax

	☐No evidence of aerodigestive injury<br><br>High-risk:<br>☐Hemodynamic instability<br>☐Progressive respiratory compromise<br>☐Tension pneumomediastinum or large pneumothorax<br>☐Suspected or confirmed aerodigestive injury
7. Conservative management (low-risk patients)	☐Admission for observation<br>☐Supplemental oxygen (high-flow if tolerated)<br>☐Continuous pulse oximetry<br>☐Analgesia<br>☐Activity restriction<br>☐Avoid positive-pressure ventilation unless essential<br>☐Avoid unnecessary invasive procedures
8. Monitoring during hospitalization	☐Serial vital signs<br>☐Daily respiratory examination<br>☐Monitor regression of subcutaneous emphysema<br>☐Monitor scrotal swelling<br>☐Repeat imaging only if clinical deterioration
9. Specialist consultation	☐Pediatric surgery / cardiothoracic surgery if deterioration or suspected injury<br>☐Urology if scrotal pain or testicular compromise
10. Discharge criteria	☐Hemodynamically stable<br>☐No respiratory distress on room air<br>☐Clinical improvement of emphysema<br>☐Tolerating oral intake<br>☐Caregivers educated
11. Caregiver education and follow-up	☐Explain benign course in stable cases<br>☐Avoid strenuous activity for 1–2 weeks<br>☐Educate on warning signs<br>☐Arrange outpatient follow-up
